# Clinical Utility of Plasma KRAS, NRAS and BRAF Mutational Analysis with Real Time PCR in Metastatic Colorectal Cancer Patients—The Importance of Tissue/Plasma Discordant Cases

**DOI:** 10.3390/jcm10010087

**Published:** 2020-12-29

**Authors:** Vincenzo Formica, Jessica Lucchetti, Elena Doldo, Silvia Riondino, Cristina Morelli, Renato Argirò, Nicola Renzi, Daniele Nitti, Antonella Nardecchia, Emanuela Dell’Aquila, Patrizia Ferroni, Fiorella Guadagni, Giampiero Palmieri, Augusto Orlandi, Mario Roselli

**Affiliations:** 1 Medical Oncology Unit, Department of Systems Medicine, Tor Vergata University Hospital, Viale Oxford 81, 00133 Rome, Italy; jess.lucchetti@gmail.com (J.L.); silviariondino2@gmail.com (S.R.); cristina.morelli89@gmail.com (C.M.); nicola.renzi86@gmail.com (N.R.); dottdanielenitti@gmail.com (D.N.); antonella.nardecchia@gmail.com (A.N.); mario.roselli@uniroma2.it (M.R.); 2 Anatomic Pathology, Department of Biomedicine and Prevention, Tor Vergata University Hospital, Viale Oxford 81, 00133 Rome, Italy; elenadoldo.ed@gmail.com (E.D.); giampiero.palmieri@uniroma2.it (G.P.); orlandi@uniroma2.it (A.O.); 3 Interventional Radiology Unit, Department of Diagnostic Imaging and Interventional Radiology, Tor Vergata University Hospital, Viale Oxford 81, 00133 Rome, Italy; renato.argiro@gmail.com; 4 Medical Oncology Department, Campus Bio-Medico University of Rome, Via Alvaro del Portillo 200, 00128 Rome, Italy; e.dellaquila@unicampus.it; 5 BioBIM (InterInstitutional Multidisciplinary Biobank), IRCCS San Raffaele Pisana, Via di Val Cannuta 247, 00166 Rome, Italy; patrizia.ferroni@sanraffaele.it (P.F.); fiorella.guadagni@sanraffaele.it (F.G.); 6 Department of Human Sciences & Quality of Life Promotion, San Raffaele Roma Open University, Via di Val Cannuta 247, 00166 Rome, Italy

**Keywords:** metastatic colorectal cancer, ctDNA, liquid biopsy

## Abstract

Background: Tumor tissue (T) mutational analysis represents the standard for metastatic colorectal cancer (mCRC); however, circulating tumor DNA (ctDNA) detected by liquid biopsy in plasma (PL) can better represent tumor heterogeneity. Methods: mCRC patients undergoing standard first-line chemotherapy with known T-KRAS/NRAS/BRAF status were enrolled in the present prospective study. PL mutations were assessed within 2 weeks before chemotherapy start with real time PCR and correlated with T status and Progression free survival (PFS). Clinical and biochemical variables including also total number of tumor lesions (TNL) and the sum of maximum diameter (SMD) of all lesions were assessed as potential predictors of T/PL discordance. RESULTS: Among 45 enrolled patients, all BRAF mutations were concordant between T and PL and there were 20% of patients RAS discordant: 9% wild type in T and mutated in PL and 11% mutated in T and wild type in PL. T mutations were significantly associated to median PFS (mPFS of 4.5, 8.3 and 22.9 months for T-BRAF mutated, T-RAS mutated, and T-wild type patients, respectively, *p* for trend 0.00014). PL mutations further refined prognosis: RAS wild type in T and mutated in PL had significantly shorter PFS than concordant RAS wild type in T and PL: mPFS 9.6 vs. 23.3 months, respectively, *p* = 0.02. Patients RAS mutated in T and wild type in PL had longer PFS than concordant RAS mutated in T and PL: 24.4 vs. 7.8 months, respectively, *p* = 0.008. At a multivariate cox regression analysis for PFS, PL mutations were independent prognostic factor superior to T analysis (HR 0.13, *p* = 0.0008). At multivariate logistic regression analysis TNL and SMD were significant predictors of discordant cases. Conclusions: PL mutational analysis allows a better prognostication than T analysis alone and could help in mCRC treatment management.

## 1. Introduction

Since 1948, when cell-free nucleic acids were first detected in human plasma [1], the interest in the molecular characteristics of the plasma cell-free DNA of cancer patients has increasingly grown in the scientific community.

Cell-free DNA is fragmented DNA found in the compartment of non-cellular molecules of the blood. In cancer patients, circulating tumor DNA (ctDNA) is released by cancer cells into the bloodstream and represents a very small fraction of the total cell-free DNA (<1%). ctDNA retains epigenetic characteristics and tumor-specific mutations of the original cancer cells and can be thus assessed in the peripheral blood, also longitudinally for patient management over time [2]. Recent studies have suggested that ctDNA can originate from multiple sources: (1) apoptotic or necrotic tumor cells from the primary tumor; (2) live tumor cells from the primary tumor; and (3) circulating metastatic tumor cells [3,4,5].

ctDNA is an ideal diagnostic tool at many time-points (pre-disease screening, early diagnosis, minimal residual disease detection after primary surgery, early recurrence diagnosis and drug resistance monitoring in the metastatic setting). 

Tumor tissue analysis remains the gold standard for cancer genotyping; however, this diagnostic procedure is often difficult to perform with inherent risk of biopsy complications, especially in relapsed and metastatic patients. Furthermore, it can be expensive and difficult to organize since it might require hospitalization resulting in significant use of healthcare resources. At times, tissue biopsy is inconclusive because of insufficient material for analysis or does not recapitulate intra-tumoral spatial and temporal heterogeneity, which would require invasive re-biopsies to be captured [6].

Moreover, the turnaround time of tumor tissue analysis is usually longer than that of ctDNA analysis, making the latter more attractive also from a technical standpoint. This would be especially useful for metastatic colorectal cancer (mCRC) patients who need rapid molecular profiling in order to receive the optimal targeted agent in a timely fashion [7]. 

Indeed, treatment of mCRC has been enriched by the introduction of biological agents targeting angiogenesis or the epidermal growth factor receptor (EGFR) signaling, that are administered in association with standard combination chemotherapy of 5-fluroruracil plus irinotecan and/or oxaliplatin [8]. Activating mutations of KRAS/NRAS (collectively named as RAS) or BRAF genes represent a major intrinsic mechanism of resistance to anti-EGFR agents and their identification is now mandatory to decide whether to use anti-EGFR antibodies or not.

In mCRC patients treated with anti-EGFR agents, RAS alteration demonstrated in liquid biopsies anticipated the radiological disease progression by approximately 10 months [9,10]. The early discontinuation of anti-EGFR therapy in case of appearance of liquid RAS mutation allows the progressive reduction in the number of circulating mutated RAS clones with the consequent reacquisition of sensitivity to the drug [11].

On the other hand, the common BRAF V600E mutation, besides being associated with anti-EGFR agent resistance, also forecasts a remarkably poor outcome in mCRC and the use of standard chemotherapy has proven of limited efficacy in this subgroup of disease [12,13]

The degree of agreement between tissue (T) and plasma (PL) mutational status is a matter of debate. In mCRC, discordant cases might be related not only to the technology used for their evaluation, but also to the patients’ features such as presence or absence of liver or lung metastases, circulating mutational fraction (defined as the fraction of mutant DNA over total cell free DNA), or tumor marker levels [14]. García-Foncillas et al. demonstrated a discordance rate of 8% between PL and T analysis in 236 mCRC patients and the discordance was higher in patients with lung metastasis [15]. Further analyses demonstrated that the maximum lesion diameter and the number of lesions have an impact on the discordance rate [16].

In the present prospective study, we assessed the clinical utility of PL liquid biopsy for KRAS, NRAS and BRAF mutation testing in mCRC using qualitative Real Time PCR (Easy PGX), in consecutive patients with known T mutational status prior to starting a standard first-line chemotherapy. We compared T and PL mutation profile and evaluated whether liquid biopsy might be useful to further stratify mCRC patient prognosis and treatment response. As RAS/BRAF mutations are both predictive and prognostic in mCRC patients treated with optimal first-line therapy [17,18], progression free survival (PFS) was chosen as a surrogate for the assessment of test usefulness. Furthermore, we investigated possible predictors of discordance between T and PL results.

## 2. Experimental Section

### 2.1. Materials and Methods

#### 2.1.1. Patients

Between September 2018 and February 2020, consecutive mCRC patients referred to the Medical Oncology Unit of Tor Vergata University Hospital (Rome, Italy), were enrolled. Inclusion criteria were age ≥18, histo-pathologically verified adenocarcinoma, and measurable metastatic disease according to the Response Evaluation Criteria in Solid Tumors (RECIST) version 1.1 not amenable to radical surgery [19]. Microsatellite instability (MSI) represented an exclusion criterion. Moreover, since the radiological tumor burden was important for the scope of the present research (see below), patients with non-measurable lesions were not eligible for the study.

Results of tissue KRAS, NRAS and BRAF were available before the start of first-line chemotherapy. Tissue assessment on either primary tumor or metastatic site was allowed, at any moment of the disease history, either at initial diagnosis or disease relapse.

Patients were treated with one of the following standard first-line regimens based on T-RAS/BRAF mutational analysis: RAS/BRAF wild type (WT) patients received panitumumab-FOLFOX (panitumumab, 6 mg/kg intravenous (IV) infusion on day 1 before FOLFOX chemotherapy regimen-oxaliplatin 85 mg/m^2^ IV on day 1, leucovorin, 200 mg/m^2^ on day 1 and 5-fluorouracil 400 mg/m^2^ IV bolus followed by 1200 mg/m^2^ IV infusion over 22 h on days 1 and 2, every 14 days); mutated patients received bevacizumab-FOLFOX (bevacizumab 5 mg/kg IV on day 1 and FOLFOX chemotherapy regimen -oxaliplatin 85 mg/m^2^ intravenous (IV) infusion on day 1, leucovorin, 200 mg/m^2^ on day 1 and 5-fluorouracil 400 mg/m^2^ IV bolus followed by 1200 mg/m^2^ IV infusion over 22 h on days 1 and 2, every 14 days). First-line chemotherapy was administered until disease progression, unacceptable toxicity or death, whichever came first.

Whole body computed tomography (CT) with or without iodinated contrast was performed every 12 weeks for tumor assessment and progressive disease (PD) was acknowledged according to RECIST 1.1 criteria. Each CT scan was carefully reviewed by a dedicated radiologist (A.R.). For each patient, the total number of metastatic lesions (TNL) and the sum of the maximum diameter of all lesions (SMD) were calculated at baseline. Of note, TNL refers to all separate lesions identified as tumor masses, which have to be distinguished from the number of metastatic sites referring to the single organs involved.

Progression-free survival (PFS) was set as the primary outcome measure and was defined as the time from chemotherapy start to PD or death from any reason. Participants who were alive but did not meet criteria for progression by the cut-off date were censored at their last evaluable disease assessment.

Common clinical variables, biochemical tests and tumor markers including carcinoembryonic antigen (CEA) and carbohydrate antigen 19.9 (CA 19.9) were also recorded before treatment and every month thereafter.

Written informed consent was obtained from all patients and the study procedures were performed in accordance with the Declaration of Helsinki and adhered to the international Good Clinical Practice guidelines.

#### 2.1.2. Molecular Analysis

##### Cell-Free DNA Extraction and Amplification

Liquid biopsy samples were collected within two weeks before first-line chemotherapy start. For cell free-DNA (cfDNA) extraction, 3 Ethylenediaminetetraacetic acid (EDTA) tubes containing 3 mL whole blood were centrifuged at 4 °C for 10 min at 3000 rpm within 1 h after blood collection. The supernatant was then transferred into 2 mL tubes, re-centrifuged at 12,000 rpm for 5 min at 4 °C and used for cfDNA extraction by MagCore^®^ Plasma DNA Extraction Kit (RBC Biosciences Corp. Taipei, Taiwan) according to manufacturer’s instructions. After extraction, cfDNA was quantified using the Qubit fluorometer ((ThermoFisher, Foster City, CA, USA), following the manufacturer’s protocol. Easy PGX Real Time PCR (DiatechPharmacogenetics Jesi, Italy) for selective mutations of KRAS and NRAS exons 2, 3 and 4 and BRAF exon 15 was performed and results analyzed using the software “Easy PGX analysis software”. The kit allowed the detection of low percentages of mutated allele in the presence of high amounts of wild-type genomic DNA by real-time amplification with sequence-specific probes marked with (5(6)-carboxyfluorescein and 6-carboxy-2′,4,4′,5′,7,7′-hexachlorofluorescein) FAM and HEX (limit of detection ranging from 0.5 to 5% depending on the mutation).

##### Tissue DNA Extraction and RAS and BRAF Mutational Status

An appropriate formalin-fixed paraffin-embedded (FFPE) tissue block was selected for each case. Three to five unstained FFPE tissue sections of the area previously selected by the pathologist (P.G.) were cut at 5 μm each for DNA extraction. DNA was obtained using the MagCore^®^ Genomic DNA FFPE One-Step Kit (Diatech-Pharmacogenetics, Jesi, Italy), according to the manufacturer’s instructions. The method is characterized by a One-Step heating, to dissolve the paraffin and, simultaneously, to lyse the tissue without dangerous substances such as xylene. DNA is extracted through cellulose co-opted with magnetic beads.

Extracted DNA was eluted in the elution buffer and then quantified by a Qubit Fluorometer (Thermofisher) using the Qubit dsDNA Assay kit, according to the manufacturer’s recommendations. Once extracted, exons 2, 3 and 4 of KRAs and NRAS and exon 15 of BRAF were amplified by Real Time PCR (Diatech Pharmacogenetics), and then genotyping of each single mutation was performed by Pyrosequencing technology (Diatech Pharmacogenetics). Using a this system, we detected the main mutations of exon 2 (codons 12, 13), of exon 3 (codons 59, 61) and of exon 4 (codons 117, 146) of KRAS and NRAS genes and main mutations of codon 600 of the gene BRAF.

### 2.2. Statistical Analysis

Patients were stratified according to concordance or discordance of tissue and plasma RAS/BRAF mutational status, and analyzed for difference of the primary outcome measure PFS with the Kaplan-Meier method and log-rank test. Hazard Ratios for PFS between patient groups were analyzed with both univariate and multivariate Cox proportional regression model and 95% confidence intervals were derived. An exploratory hypothesis-generating multivariate Cox regression analysis was run including both tissue and plasma mutational status, as well as known prognostic factors. This analysis was not performed to specify a prognostic model. Instead, it was performed to assess the relative contribution of important covariates to PFS estimation in our patient cohort. For this reason, no variable reduction/pre-selection (such as univariate pre-selection) was carried out and no strict rule for the number of variables to be included in the analysis was applied.

Since discordant mutational status was thought to be clinically relevant, a multivariate logistic regression analysis including baseline patient characteristics was performed to identify determinants of discordant results. A Least Absolute Shrinkage and Selection Operator (LASSO) approach was used to select the most significant factor predicting mutational discordance.

For the logistic regression analysis, continuous variables were optimally dichotomized by means of ROC curve analysis. Differences in variables between concordant and discordant cases were assessed with the chi-square statistics and Odds Ratio calculation.

All analyses were performed with R software version 3.6.0 (Vienna, Austria—http://www.R-project.org/). Tests were considered statistically significant for two tail *p* values <0.05.

## 3. Results

### 3.1. Patients’ Characteristics

Between September 2018 and February 2020 45 mCRC patients were enrolled (median age 67 y, 16 females, 29 males). Patients’ characteristics are summarized in Table 1). Karnofsky Performance Status (KPS) was 100 in 84% of cases. Only 7 patients had KPS ≤ 80 at the moment of the liquid biopsy, and only for two patients a KPS deterioration was noted since the initial tumor diagnosis.

More than half of patients had liver metastasis (30 out of 45 patients, 66.67% of total), and 11 had liver limited disease (24.45%). Median TNL was 4 (range 1–56), and median SMD expressed in mm was 122 (range 25–1687 mm).

According to tissue mutational status, 6 patients were BRAF mutated (13%), 15 RAS mutated (34%), and 24 RAS/BRAF wild type (53%). As per protocol, 21 patients (47%) received FOLFOX-bevacizumab (RAS or BRAF mutated patients) and 24 (53%) FOLFOX-panitumumab (RAS/BRAF wild type).

In 62% of cases, tissue sampling for mutational assessment had been performed within the year preceding the liquid biopsy date and first-line chemotherapy start. More ancient tissue sampling (>1 year) was mainly due to metachronous metastatic relapse of initially localized colon cancer. None of the patients were exposed to anti-EGFR agents in the interval time between tissue sampling and liquid biopsy. Tissue source was surgically-removed primary tumor, colonoscopic biopsy and biopsy of a metastatic lesion (mainly liver metastasis) in 16% (*n* = 7), 55% (*n* = 25) and 29% (13) of cases, respectively.

### 3.2. Survival Analysis

**Figure 1:**
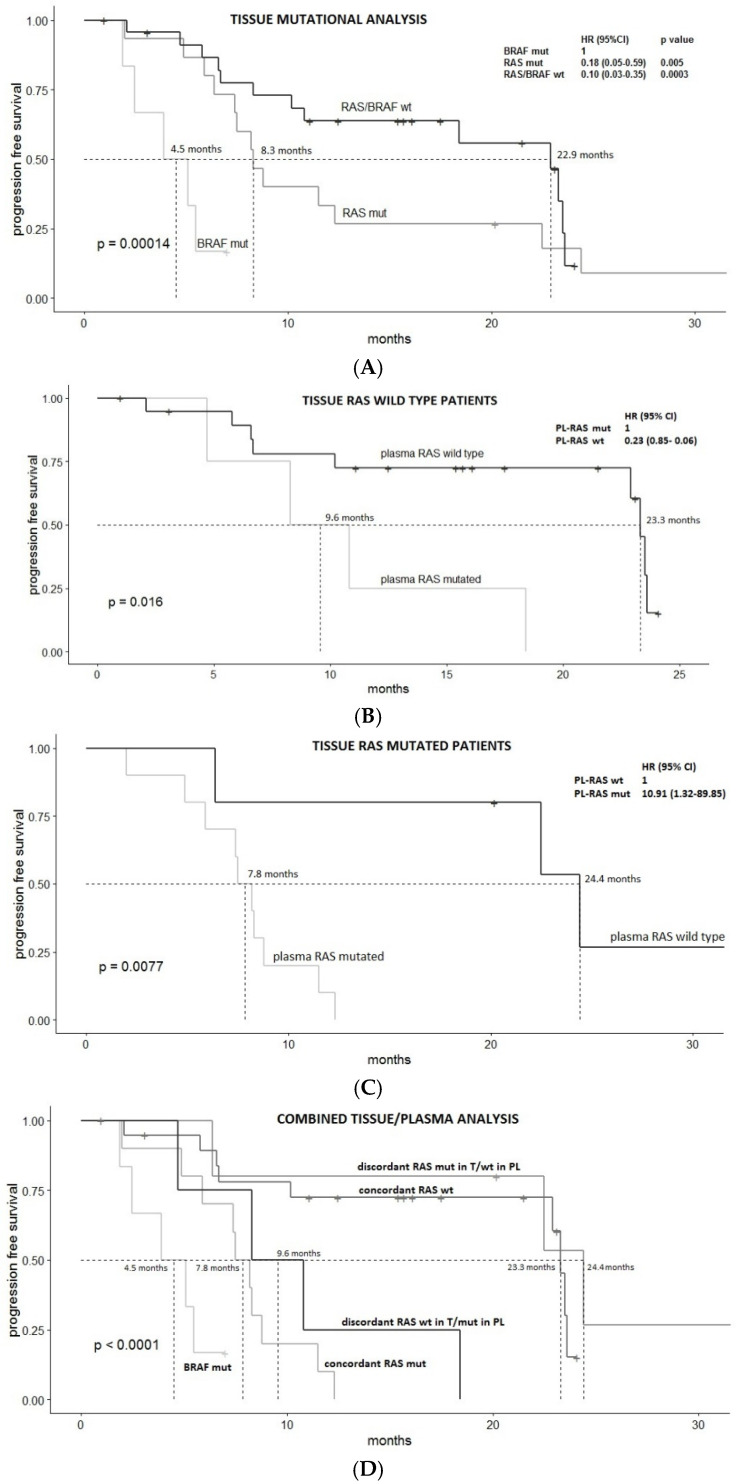
Progression free survival (PFS) analysis. (**A**): PFS according to tissue mutational analysis; (**B**): PFS in the tissue RAS wild type group according to the result of the liquid biopsy, (**C**): PFS in the tissue RAS mutated group according to the result of the liquid biopsy; (**D**): PFS in the five subgroups identified by the combined analysis of tissue and liquid biopsy. mPFS is indicated. *p* value in the bottom left angle refers to *p* value for trend for (**A**,**D**). BRAF V600E mutations were always consistently found both in plasma and in tissue (BRAF mut subgroup).

Tissue mutational status was confirmed to have a statistically significant prognostic value, with median PFS (mPFS) of 4.5, 8.3 and 22.9 months for tissue BRAF V600E mutated, tissue RAS mutated, and tissue RAS/BRAF wild type mCRC patients, respectively, *p* = 0.00014 (taking BRAF mutated group as reference, Hazard Ratio, HR 0.18, 95%CI 0.05–0.59, *p* value 0.005 and HR 0.10, 95%CI 0.03–0.35, *p* value 0.0003, for tissue RAS mutated and tissue RAS/BRAF wild type patients, respectively) (Figure 1A).

According to liquid biopsy compared to tissue RAS/BRAF mutational status, we found 100% of concordance for BRAF V600E mutation and 9 discordant cases for RAS mutation. Hence, the combined mutational analysis of tissue and plasma identified the following 5 subgroups: patients BRAF mutated concordant (*n* = 6), patients RAS wild type (WT) in tissue (T) and mutated in plasma (PL) (RAS WT-T/MUT-PL discordant) (*n* = 4), patients RAS mutated in T and wild type in PL (RAS MUT-T/WT-PL discordant) (*n* = 5), patient RAS mutated in both T and PL (RAS MUT concordant) (*n* = 10), patient RAS wild type in both T and PL (RAS WT concordant) (*n* = 20).

Plasma RAS mutational status was significantly associated with PFS in both tissue RAS wild type and mutated patients: mPFS of concordant vs. discordant cases was 23.3 vs. 9.6 months, respectively in tissue RAS wild patients (HR 0.23 (95% CI 0.85–0.06), *p* = 0.02) (Figure 1B); and 7.8 vs. 24.4 months, respectively in tissue RAS mutated patients (HR 10.91 (95% CI 89.85–1.32), *p* = 0.008) (Figure 1C).

Figure 1D reports survival curves of the five subgroups. PFS for RAS MUT-T/WT-PL was similar to that of RAS wild type concordant cases (*p* value 0.399) and PFS for RAS WT-T/MUT-PL was similar to that of RAS mutated concordant cases (*p* value 0.547).

In an exploratory multivariate cox regression analysis including known prognostic factors such as performance status, CEA and tumor sidedness, liquid mutational status was confirmed to be an independent prognostic factor superior to tissue mutational status (*p* = 0.0008) (Table 2). Of note, patients with BRAF mutation were excluded from the analysis.

### 3.3. Predictors of Discordant Cases

Given the relevance of RAS discordant cases, a multivariate logistic regression (MLR) analysis with least absolute shrinkage and selection operator (LASSO) approach was performed to identify the most significant predictors of liquid/tissue discordance in both subgroups of tissue RAS mutated and tissue RAS wild type patients.

The analysis included the following 23 clinical and biochemical variables recorded at baseline: type of tissue source, tissue collection-liquid biopsy interval time, primary sidedness, resection of primary tumor, total number of tumor lesions (TNL), total tumor burden expressed in mm (SMD), presence of liver metastases, age, sex, albumin, red blood cells, hemoglobin, white blood cells, lymphocytes, neutrophils, platelets, d-dimer, C reactive protein, Charlston comorbidity index, CEA, CA19.9, KPS basal score, smoking habit.

Because of the small size of the patient cohort, individual ROC curve analysis was first performed to optimally dichotomize continuous variables.

**Figure 2:**
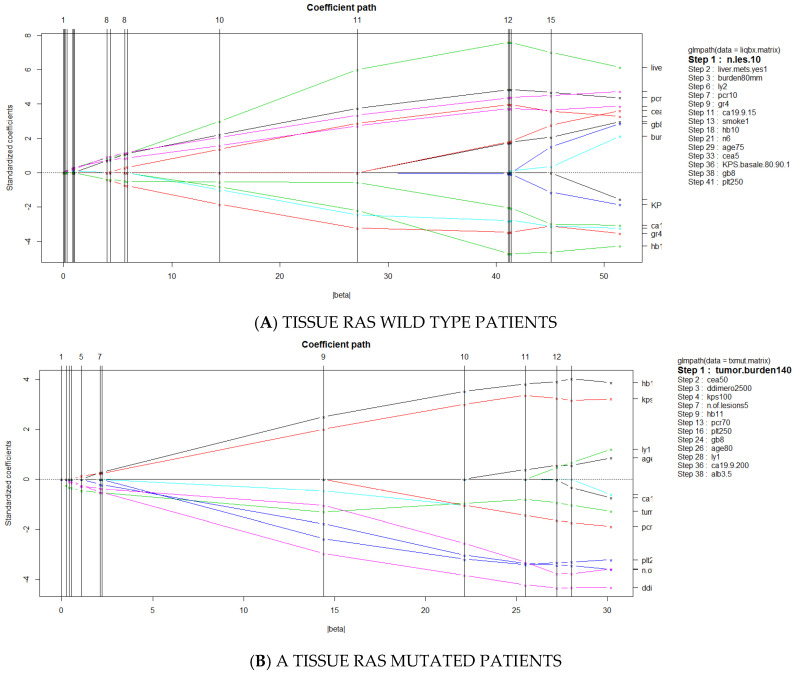
Path of Multivariate Logistic regression analysis with Least Absolute Shrinkage and Selection Operator (LASSO) approach for RAS discordance.

According to MLR with LASSO, TNL was found to be the most significant predictor of discordance among tissue RAS wild patients (*p* = 0.05) (Figure 2A), and SMD the most significant predictor of RAS discordance among tissue RAS mutated patients (*p* = 0.02) (Figure 2B). Of note, SMD and TNL were superior to source of tissue biopsy (biopsy vs. surgical material) and interval time from tissue to plasma collection (< vs. >1 year) in predicting discordance.

**Figure 3:**
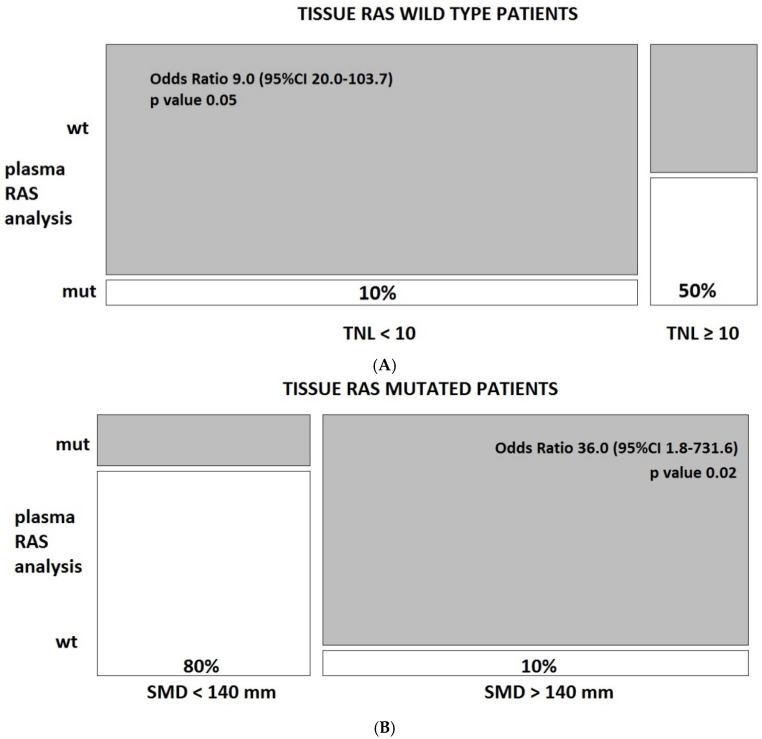
Mosaic plot of risk of discordance according to total number of tumor lesions (TNL) and sum of the maximum diameter (SMD) of all lesions in tissue RAS wild type (**A**) (*n* = 24) and mutated patients(**B**) (*n* = 15).

The absolute risk of discordance was 50% vs. 10% in patients with ≥10 vs. <10 total number of lesions in the tissue RAS wild type subgroup (Odds Ratio 9.0, *p* = 0.05), and was 80% vs. 10% in patients with <140 vs. >140 mm of overall SMD in the tissue RAS mutated subgroup (Odds Ratio 36.0, *p* = 0.02) (Figure 3).

## 4. Discussion

In the present prospective study, we demonstrated that baseline plasmatic testing of ctDNA RAS/BRAF mutation using Easy PGX Real Time PCR offers meaningful additional information in mCRC patients starting a standard firs-line chemotherapy. In our cohort, plasma RAS assessment was even superior in terms of PFS prediction as compared to tissue RAS assessment (*p* value at multivariate Cox regression analysis, 0.0008 and 0.702, respectively). BRAF mutation detected in the plasma had a perfect concordance with tissue analysis and was confirmed to be associated with the worst PFS (mPFS 4.5 months) [20].

Although the authors acknowledge the small size of the study cohort and that caution should be taken also in interpreting significant results, useful information can be derived from the analysis of predictors of discordant cases for KRAS and NRAS genes. In subjects formally labelled as RAS wild type at the tissue analysis, a high percentage of patients with circulating RAS mutated clones was found when more than 10 tumor lesions were detected at the pre-chemotherapy CT scan (50% of cases). This is probably due to both spatial and temporal heterogeneity since tissue was often collected many months before the disease progression requiring a first-line chemotherapy or on cell clones (mainly from the primary site) that were not representative of the entire disease, especially when a relevant number of metastatic lesions are observed (i.e., >10). We therefore strongly recommend a ‘liquid re-profiling’ when more than 10 distinct tumor masses are displayed at the CT scan before starting a first-line treatment containing an anti-EGFR antibody. Only 13 patients in our cohort had tissue mutational assessment in the metastatic site (mainly the liver), and further assessment of primary vs. metastasis spatial discordance could not be carried out.

On the other hand, an even higher discordance (80%) was found among tissue RAS mutated patients with a low tumor burden (overall <140 mm as measured by SMD). The most probable explanation of this finding is the higher false negativity rate of liquid biopsy testing observed when a specific mutation present in the tumor tissue is analysed in the plasma of patients with low tumor burden. Modern techniques with improved sensitivity would probably detect circulating mutations also in case of low mutant allele fraction (MAF) [21,22,23].

From a biological point of view, it is possible that in some metastatic patients, given the increasing reduction of limit of detection (LoD) for RAS mutation of new generation genotyping techniques, a disease formally labelled as tissue mutated is still constituted for the vast majority of RAS wild type clones, with less aggressive behaviour, and more sensitive to anti-EGFR agents. Liquid biopsy would represent, in this case, the ‘clinically meaningful’ disease with mutated clones confined in limited spatial niches and not shedding in the bloodstream. Interestingly, in this subgroup, the mean diameter of the lesions was higher among the discordant cases (median 34 mm, range 20–100 mm) than among concordant cases (median 29 mm, range 15–80), indicating that this hypothesis might be particularly true in patients with few lesions of big dimensions.

Moreover, the organs involved by metastatic dissemination might be of relevant importance. Among discordant patients with RAS tissue mutated and plasma wild type, only 2 out of 5 had liver metastases. The absence of liver metastasis is a condition more often associated with low cfDNA release in peripheral blood. In fact, as reported in other works using even more sensitive techniques, the absence of liver metastases was the main clinical factor associated with inconclusive ctDNA results [24].

Various technologies are available for testing known mutations in ctDNA in mCRC. They are classified into PCR-based or sequencing-based technologies (NGS). NGS allows deep sequencing of amplicons and, while the PCR-based methods can detect specific, already-known mutations, NGS has the advantage of detecting novel mutations in addition to the known ones [25,26].

Methods based on real-time PCR, widely used in clinical research, have a limit of detection (LoD) of 0.5–0.1%. Because ctDNA exists at low levels in plasma, more sensitive detection methods, such as the allele-specific quantitative PCR-based Intplex technology, emulsion PCR techniques (such as ddPCR), and the Beads, Emulsion, Amplification and Magnetics (BEAMing) have been developed.

These methods have a limit of detection of 0.01–0.001%, resulting in a higher ability in detecting low frequency mutations [27]. However, the ability to diagnose rare RAS mutant subclones has uncertain clinical significance and might lead to an over-diagnosis of anti-EGFR resistance [28].

Although ctDNA typically constitutes only a small portion (<1%) of the total cfDNA [29,30], it is regarded as a reliable tool in oncology, offering a non- invasive method to evaluate patients’ genomic profiles and consenting repeated evaluations during the course of the disease.

Moreover, its use, in contrast to the analysis of tumor biopsy samples, allows an overview of tumor heterogeneity and evolution. This last point is of the outmost importance, given that tumor heterogeneity, which characterizes cancers in an advanced stage [31], limits the use of tissue biopsy for reliable cancer sequencing [29].

The same patient might display both an intra-tumoral heterogeneity (in which different areas of the same tumor mass have different genetic profiles), and inter-metastatic heterogeneity when multiple distinct metastatic lesions are present [32].

Our results highlight the importance of the information derived from discordant cases. The vast majority of the studies on ctDNA underline the good level of concordance between the two methods and, indeed in our population, we have observed a significant level of concordance of tissue/plasma mutations (80%), consistent with data reported in the literature [33,34,35]. However, we also suggest that discordant cases might be of clinical relevance, instead of just indicating false negative or false positive errors.

In some reports, discordant findings are explained by the temporal heterogeneity and the possible acquisition of mutation during targeted treatment with anti-EGFR agents [36]. However, none of the patients enrolled in our study received an anti-EGFR treatment.

A prospective phase II clinical trial (PROSPECT-C) of single agent anti-EGFR therapy in patients with RAS WT metastatic colorectal cancer (mCRC) found that almost half of patients harbored pre-existing alterations in the RAS pathway, detectable at baseline cfDNA evaluation. These patients had a significantly lower PFS and OS as well as a trend toward lack of response compared with patients without baseline RAS alterations on liquid biopsy [37].

These data are in agreement with those of Vandeputte et al., who observed that mCRC patients with an early decline in mutant DNA fraction during regorafenib and TAS-102 treatment had a longer mPFS than those displaying a persistently high mutant fraction [38].

In the Italian CRICKET (**C**etuximab **R**echallenge in **I**rinotecan-pretreated m**C**RC, **K**RAS, NRAS and BRAF Wild-type Treated in 1st Line With Anti-**E**GFR **T**herapy) study 27 mCRC patients, with an initial tissue wild type RAS/BRAF status and the subsequent development of acquired resistance during first-line anti-EGFR-based therapy, were re-challenged with irinotecan plus cetuximab in third line after an anti-EGFR-free second-line. At the re-challenge baseline, mutational assessment of ctDNA by droplet digital polymerase chain reaction (ddPCR) and next-generation sequencing (NGS) predicted a greater efficacy of irinotecan-cetuximab re-challenge [39].

All these data show how liquid biopsy can be used to optimize therapy management in mCRC and to early detect drug resistance.

We must acknowledge the following limitations of our study: (1) the low sensitivity of the technique used (LoD 0.5–5%), (2) the small sample size, (3) the absence of serial liquid biopsies during treatment. Despite these limitations, our study is in line with the recently released consensus statement of the Colon and Rectal-Anal Task Forces of the United States National Cancer Institute on the utility of ctDNA in the management of mCRC patients, with the ultimate goal to include it in routine clinical practice [40]. Extension of the present study to enroll a higher number of patients is underway. Prospective comparison of real time PCR to other more sensitive techniques is also being considered.

## 5. Conclusions

Liquid biopsy for the assessment of RAS and BRAF mutations using available kits of real time PCR is feasible and effective in clinical management of mCRC patients. Even if the emergence of circulating RAS mutated clones has been previously correlated with anti-EGFR antibody acquired resistance [41], the present study confirms that ctDNA assessment should be taken into consideration also at the baseline in certain chemotherapy-naïve patients.

Together with clinical information such as the number and dimension of total metastatic lesions, liquid biopsy seems associated with a more informative prognostic value than tissue mutational status alone. Larger sample size is required to confirm these results. Future studies should evaluate the extent by which baseline ctDNA mutational analysis would influence the choice of targeted agents in mCRC.

## Figures and Tables

**Table 1:** Patients characteristics. KPS: Karnosky Performance Status. TNL: total number of tumor lesions. SMD: sum of maximum diameter of all measurable lesions in mm.

Characteristic	% of Patients (Total *n* = 45)	Characteristic	% of Patients (Total *n* = 45)
**Median Age (years, range)**	67 (41–89)	**Median Karnofsky Performance Status (KPS) score (range)**	100 (60–100)
**Gender**		**Sidedness**	
Female	36% (16)	Right	22% (10)
Male	64% (29)	Left	78% (35)
**Metastasis**		**Mutational status in tissue**	
Liver only	24% (11)	BRAF mutation	13% (6)
Liver and others	67% (30)	RAS mutation	34% (15)
Non-liver only	9% (4)	RAS/BRAF wild type	53% (24)
**Tumor burden**		**Treatment**	
TNL	4 (1–56)	FOLFOX-bevacizumab	47% (21)
SMD	122 mm (25–1687)	FOLFOX-panitumumab	53% (24)
**Primary Resection**		**Tumor markers**	
Yes	73% (33)	Carcinoembryonic antigen (CEA) (median ng/mL, range)	599 (0.62–5580)
No	27% (12)	CA19.9 (median ng/mL, range)	214 (1–19,032)

**Table 2:** Multivariate Cox-regression analysis for PFS. In bold variables with independent prognostic value are reported. Coef: beta coefficient, HR: hazard ratio, Se: standard error, Z: z-statistic.

Covariate	Coef	HR	Se (Coef)	Z	*p*
CEA	0.0001	1.0001	0.0002	0.5390	0.5899
Tissue mutational status	0.1897	1.2088	0.4957	0.3830	0.7020
**Plasmatic mutational status**	**−1.9731**	**0.1390**	**0.5912**	**−3.3370**	**0.0008**
**Sideness left vs. right**	**−0.0118**	**0.9883**	**0.5185**	**−0.0230**	**0.9818**
**KPS**	**−0.0743**	**0.9284**	**0.0281**	**−2.6440**	**0.0082**

## Data Availability

MDPI is committed to supporting open scientific exchange and enabling our authors to achieve best practices in sharing and archiving research data. We encourage all authors of articles published in MDPI journals to share their research data. More details in section “MDPI Research Data Policies” at https://www.mdpi.com/ethics.
